# tmQMg* Data Set: Excited State Properties of 74k Transition Metal Complexes

**DOI:** 10.1021/acs.jcim.5c01958

**Published:** 2025-10-24

**Authors:** Hannes Kneiding, David Balcells

**Affiliations:** Hylleraas Centre for Quantum Molecular Sciences, Department of Chemistry, 6305 University of Oslo, P.O. Box 1033, Blindern, 0315 Oslo, Norway

## Abstract

The application of machine learning approaches to meaningful problems in chemistry and materials science is still challenged by the limited availability of data. In order to close this gap, we report the tmQMg* data set, which provides excited state properties for 74k mononuclear transition metal complexes extracted from the Cambridge Structural Database. All properties were computed at the TD-DFT ωB97xd/def2SVP level of theory. The strongest electron excitations in the ultraviolet, visible, and near-infrared ranges are included, together with the wavelengths and intensities of the first 30 excited states. Further, natural transition orbitals were computed for the strongest excitations in the visible range to determine the nature of the associated charge transfers. By computing the TD-DFT spectra in both gas phase and acetone, we quantified solvatochromic effects, which are also provided with the data set, in terms of both wavelength shifts and intensity changes. The tmQMg* data set will enable the development of discriminative and generative artificial intelligence models with respect to absorption spectra, charge transfer character, and solvatochromism, enabling novel advances in the field of transition metal photochemistry.

## Graphical abstract

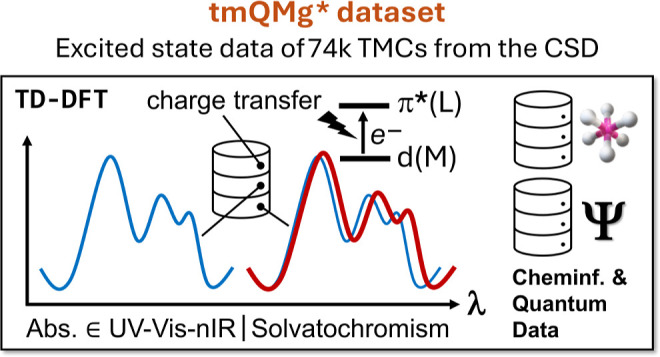

## Introduction

Artificial intelligence (AI) is quickly becoming a widespread tool across all scientific disciplines including chemistry and materials science.^1^ By fitting to reference data, discriminative machine learning (ML) models learn to predict properties of molecules and materials based on sparse feature sets. Their computational efficiency makes ML approaches especially attractive for high-throughput studies in molecular and materials design where large numbers of compounds are investigated in order to identify promising candidates for specific applications.^2,3^ Compared to theoretical reference methods, such as density functional theory (DFT), a speed-up of up to 5 orders of magnitude can be achieved at no significant loss of accuracy.^4,5^ Furthermore, generative AI approaches can be used in an inverse design framework where models are trained to generate new compounds that are similar to the reference data.^6,7^ Both approaches have received a lot of attention particularly from the chemistry and materials science communities, promoting their rapid advancement.^8,9^

Despite the empirically proven potential of AI in accelerating the materials discovery pipeline, its application to interesting, real-world problems is still challenged by the limited availability of appropriate and sufficiently large data sets.^10^ Due to the prohibitively high cost associated with experimental data sets, most large data sets are based on computations oftentimes obtained with quantum chemistry methods.^11^ While still significant, the reduced cost of these approaches, and their easier design and scale-up, allowed for the creation of large data sets that are fit for the application of ML methods. However, most of the existing computational data sets are limited to either organic chemistry (for example: QM^12–14^ or GDB^15,16^ data set series) or materials science (for example: Materials Project,^17^ Open Quantum Material Database^18^), for which extensive work has been conducted before. The more recent OMol25 data set integrated multiple molecular sets of various nature.^19^

Lately, there has been a surge of interest in metal-containing systems such as transition metal complexes (TMCs) and metal–organic frameworks (MOFs) for diverse applications in catalysis and renewable energies. This is reflected in the creation of a significant number of data sets for both TMCs (tmQM series^20–22^ ) and MOFs (CoRE data set^27–29^ ). In particular, for TMCs, the available data sets have supported the development of ML models for the prediction of their electronic properties^21,23,30–33^ as well as their (un)­conditional generation.^22,34,35^ However, since most data sets only contain ground state properties, the development of models for photochemistry and, in general, applications to nonadiabatic processes involving excited states,^36–38^ is significantly impeded. Data sets for excited state properties including absorption spectra and charge transfer behavior are rare, though available for small molecules.^39^ Nonetheless, such properties are of particular interest for TMCs due to their potential use as photocatalysts,^40^ molecular sensitizers in solar cells,^41^ optical switches in optoelectronic devices,^42^ or photosensitizers in cancer phototherapy.^43^

In this work, we report the tmQMg* data set, providing electron excitation data for 74k mononuclear TMCs. The data was computed in the Saga supercomputer of the Norwegian high-performance computing cluster at the TD-DFT­(ωB97xd/def2SVP) level of theory^44–46^ using Gaussian 16, Revision C.01.^47^ tmQMg* belongs to the tmQM data set series (Figure 1),^20–22^ and it is thus based on a large set of TMCs that are experimentally known and reported in the Cambridge Structural Database^48^ (CSD, 2024 release), often used for data-driven approaches.^49,50^ With all 30 transition metals from the 3d, 4d, and 5d series present, and an underlying pool of more than 35k unique ligands, tmQMg* reflects the vast diversity of the TMC chemical space known to date. The tmQMg* data set will enable the training of both discriminative and generative AI models with respect to electron excitations in TMCs and their charge transfer nature, light absorption intensity and broadness over the whole UV–vis-nIR range, and solvatochromic effects. These models will accelerate the discovery of photoactive TMCs. tmQMg* is openly available under the CC BY 4.0 License.

**Figure 1:**
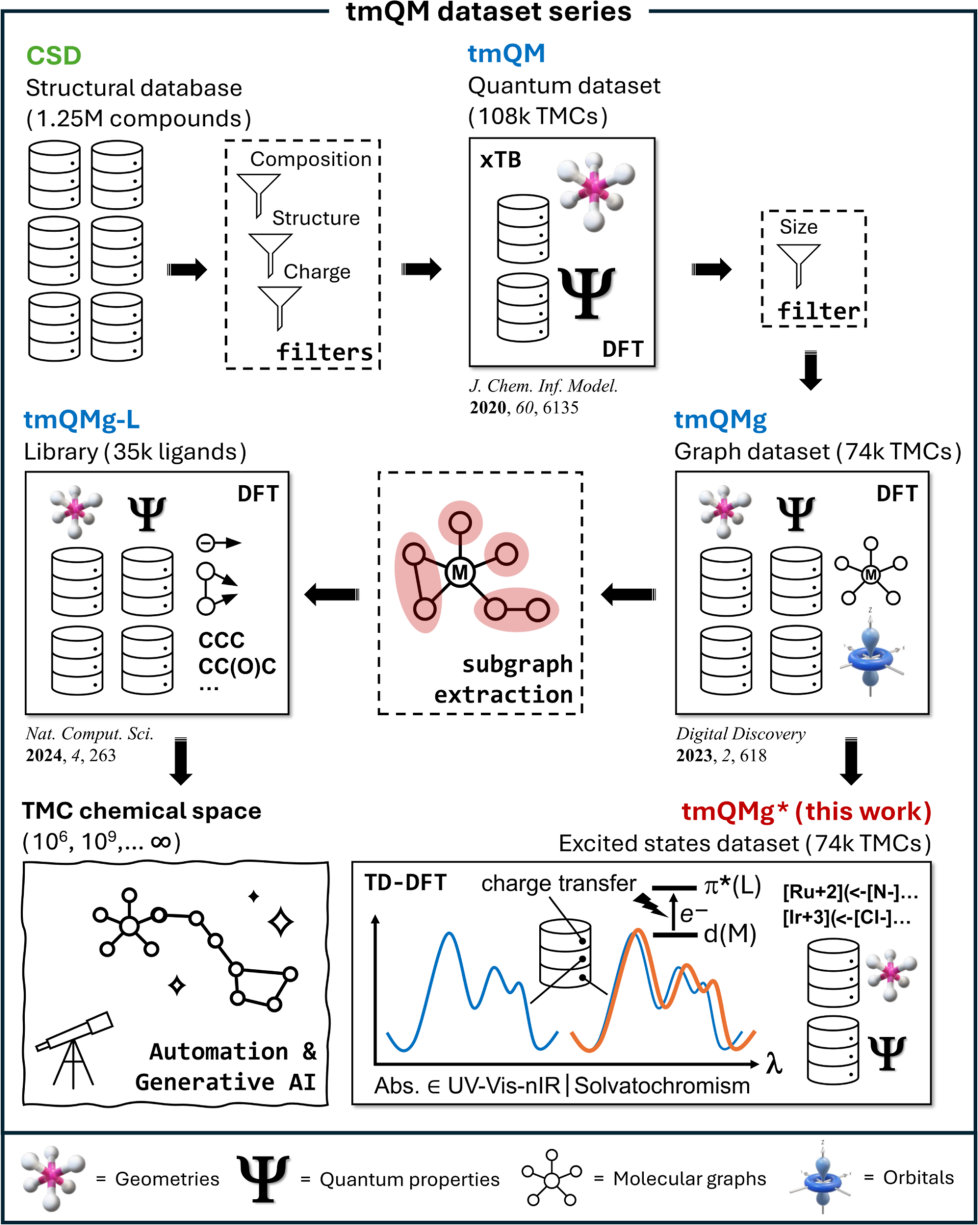
tmQMg* in the context of our founding work on the tmQM data set series.^20–22^ Further extensions have been reported by other research groups.^23–26^

## The tmQMg* Data Set

The tmQMg* data set was derived from single point TD-DFT­(ωB97xd/def2SVP) calculations^44–46^ on the 74,555 TMCs of the parent tmQMg graph data set, in which geometries were already optimized at the DFT­(PBE/def2SVP) level.^21^ This level of theory was chosen due to the relevance of including long-range corrections in TD-DFT calculations. The Tamm-Dancoff approximation was not used. 99.6% of these calculations converged successfully, yielding the electron excitation data of the 74,281 TMCs included in tmQMg*. Natural transition orbitals (NTOs) were also computed at the same level of theory to determine the charge transfer nature of the transitions. Further, solvatochromic effects were quantified by computing all TD-DFT data in two sets: one in gas phase and one in acetone, using the implicit SMD solvation model.^51^

In the frame of the transition metal quantum mechanics series, which includes the tmQM,^20^ tmQMg,^21^ and tmQMg-L^22^ data sets (Figure 1), there is plenty of additional data available for each TMC in tmQMg*: quantum properties at different xTB and DFT levels of theory (energies, geometries, HOMO–LUMO gaps, polarizabilities, atomic charges, and dipole moments, among others),^20^ quantum-informed molecular graphs for deep learning models (directed and undirected),^21^ and a substantial portion of the ligands forming this TMC space,^22^ including the ligand charges and metal-coordination anchors needed to design, generate, and explore massive combinatorial spaces. The TMC SMILES based on the recent work of Jensen and co-workers^52^ are also available. In particular, we used the Hückel approach, in which this method provides the graph representation of the TMC needed to formulate its SMILES string. All TMCs were extracted from the 2024 release of the CSD, constraining the charge to the {−1, 0, +1}*e* set.^20^ This constraint, was originally implemented to avoid issues in the quantum chemistry calculations caused by smaller and higher charges. All TMCs in tmQMg* have an even number of electrons and all TD-DFT calculations on them were carried out in the closed-shell singlet state.

### Chemical Distributions

Figures 2 and 3 show the chemical composition of the tmQMg* data set in terms of element distributions. The 3d, 4d, and 5d transition metals appear represented to a similar extent and, for all three series, the late metals are clearly more present than the early (Figure 2). TMCs with metal centers from groups 8, 9, and 10 are the most abundant, likely due to their popularity in impactful applications like catalysis. In this regard, and despite the computational nature of the tmQMg* data set, its CSD origin reflects, and is thus biased by, the interest of the research community in TMC chemistry over a period of 60 years (1965–2024). It is important to note that this can introduce significant biases if the tmQMg* data set is used to train ML models for the prediction of properties related to photochemistry. Transferable models across the transition metal series will likely require data augmentation strategies for the early transition metals, where only group 4 appears to be well represented and balanced over the three series.

**Figure 2:**
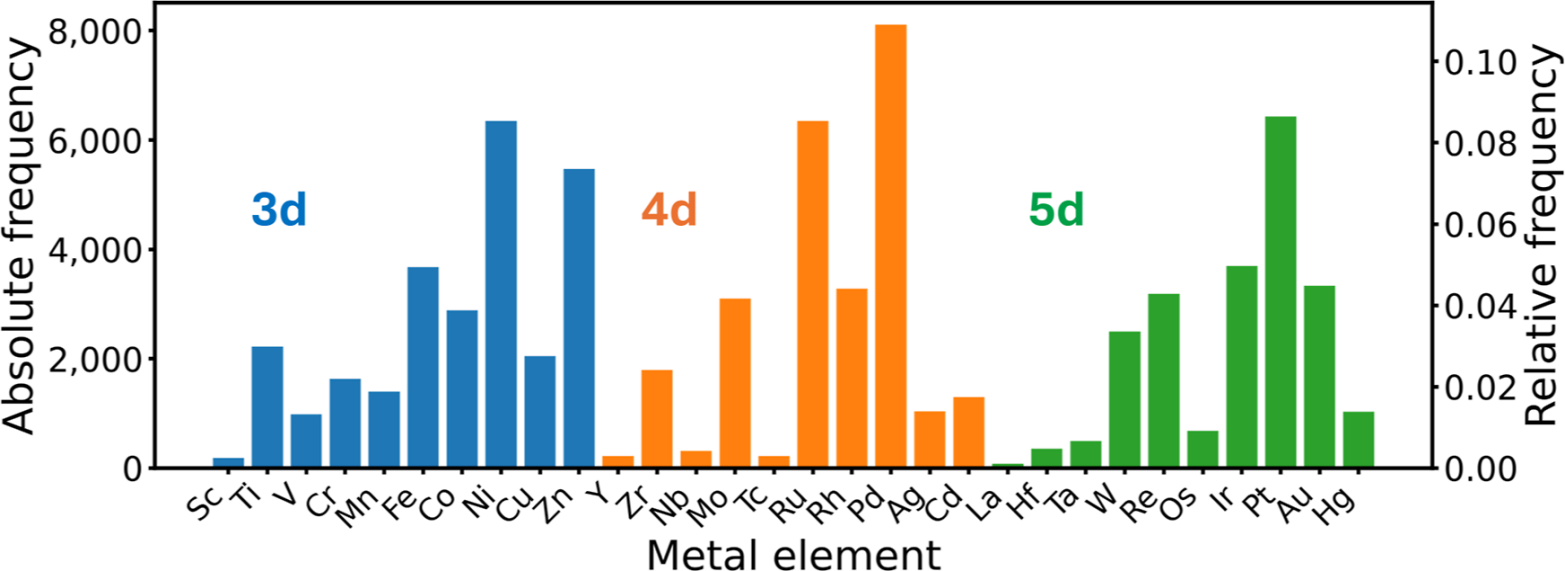
Distribution of the transition metal elements in the tmQMg* data set.

**Figure 3:**
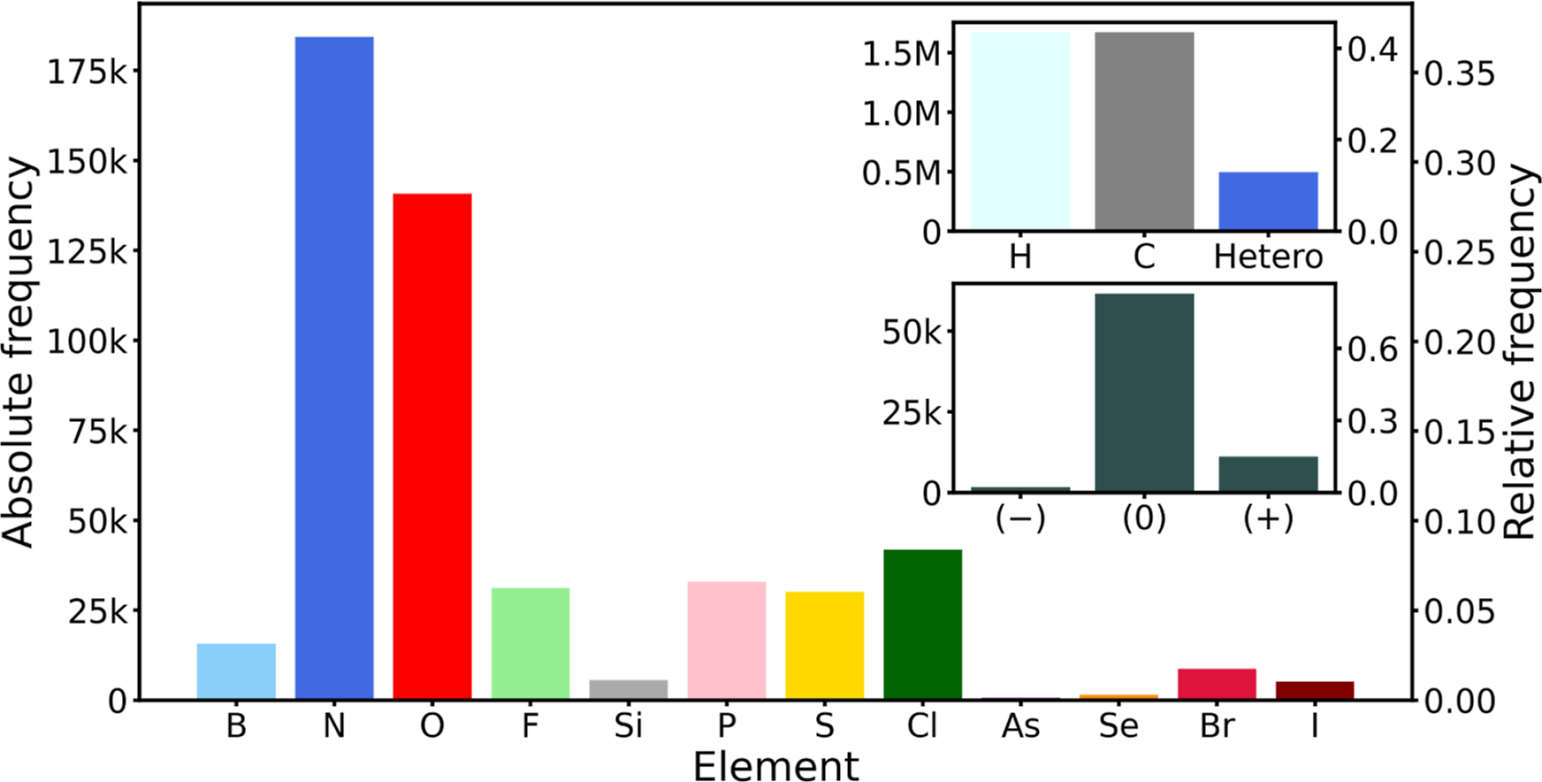
Distribution of the nonmetal elements in the tmQMg* data set. The insets show the abundance of C and H relative to the heteroelements, and the TMC charge distribution.

In line with the prevalent organic nature of the ligands, C and H are the most abundant elements in the data set, followed by N and O, which are common metal anchors, and the less abundant P and Cl associated with phosphine complexes and metal-chloride salts (Figure 3). Regarding charges, the distribution is strongly dominated by neutral TMCs. Charges larger or smaller than +1 and −1, respectively, were excluded by the filters used to extract the TMCs from the CSD into the parent tmQM^20^ and tmQMg^21^ data sets. To some extent, this limits the value of tmQMg* in photoredox chemistry involving highly charged species, suggesting an interesting (as well as challenging and expensive) direction for data augmentation.

### Electron Excitations

For each TMC, the TD-DFT calculations yielded the first 30 electron excitations, for which both the wavelength (λ) and the oscillator strength (*f*) were extracted and included in the data set. Table 1 provides a systematic list of all TD-DFT properties in tmQMg*, including the column labels used in the tabular CSV file in the data repository.

**Table 1:** TD-DFT­(ωB97xd/def2SVP) Properties in the tmQMg* Dataset

Column name in CSV	description
lambda_i^a^	Wavelengths, λ, in nm; i ∈ [1–30] is a counter
f_i^a^	Oscillator strengths, *f*, unitless; i ∈ [1–30] is a counter
lambda_max^a^ ^,^ ^b^	Wavelengths having the largest *f*, λ_max_, in nm
f_max^a^ ^,^ ^b^	*f* associated with λ_max_, *f*_max_, unitless
sigma^a^ ^,^ ^b^	Band broadness, σ, in nm
M_contribution^a^ ^,^ ^c^	The relative contribution (%) of the metal to the NTO orbitals involved in the strongest Vis excitation
L_contribution^a^,^c^	The relative contribution (%) of the ligand(s) to the NTO orbitals involved in the strongest Vis excitation
transition_nature_vis^a^	The charge transfer nature of the strongest Vis excitation. Categorical over the {ddT, LMCT, MLCT, LLCT} set
lambda_delta	Solvatochromic wavelength shift, Δλ, in nm
f_delta	Solvatochromic oscillator strength change, Δ*f*, unitless
vis_to_vis	Vis to Vis shift. Boolean, True or False
uv_to_vis	UV to Vis shift. Boolean, True or False
nir_to_vis	nIR to Vis shift. Boolean, True or False
vis_to_uv	Vis to UV shift. Boolean, True or False
vis_to_nir	Vis to nIR shift. Boolean, True or False
bathochromic	Δλ > 0 from gas phase to acetone. Boolean, True or False
hypsochromic	Δλ < 0 from gas phase to acetone. Boolean, True or False
hyperchromic	Δ*f* > 0 from gas phase to acetone. Boolean, True or False
hypochromic	Δ*f* < 0 from gas phase to acetone. Boolean, True or False

a Determined for gas phase and in acetone, denoted by the suffixes _gasphase and _acetone, respectively.

b Determined for the three ranges UV, Vis, and nIR, denoted by the suffixes _uv, _vis, and _nir, respectively.

c Determined for the occupied and virtual transition orbitals, denoted by the suffixes _occupied and _virtual, respectively.

Based on the value of λ, the ultraviolet (UV), visible (Vis), and near-infrared (nIR) regions of the spectrum were defined as follows:

- UV: λ < 350 nm
- Vis: 350 nm ≤ λ ≤ 825 nm
- nIR: λ > 825 nm

Most electron excitations lie in the UV region of the spectrum, followed by the Vis and nIR regions. The gas phase/acetone percentages are 90/92% (UV), 9/8% (Vis), and <1/1% (nIR). The prevalence of UV excitations, both in number and intensity, can be associated with the π → π* transitions, which, in a TMC chemical space, can originate not only from the isolated ligands, but also from diverse metal–ligand and ligand–ligand moieties.

Figure 4 shows the averaged spectra over the entire tmQMg* data set. In the gas phase, and considering all electron excitations, the spectrum has a prominent maximum in the UV region, at λ = 185 nm. Moving toward longer wavelengths, light absorption decays and, after forming a shoulder over the UV|Vis limit, it yields two lower peaks at λ = 625 nm, within the Vis, and at λ = 836 nm, within the nIR. When averaged after applying the *f* > 0.01 threshold, the spectrum becomes more balanced, with these three peaks appearing at similar λ values (185, 645, and 835 nm), plus an additional one in the Vis region (375 nm) that can be related to the aforementioned shoulder. The difference between the two spectra reflects the underlying structure of the data. For example, compared to the UV range, the electron excitations in the Vis are less numerous and fewer of them have high intensity. In both spectra there is a small but significant bathochromic shift, as reflected by the relocation of the absorption peaks to longer wavelengths. For example, in the averaged *f* > 0.01 spectra, the four peaks, ordered from UV to nIR, undergo Δλ shifts of 4.7, 10.3, 37.2, and 16.6 nm, when, respectively, moving from gas phase reference to acetone. This effect can be related to the larger polarization of the excited states often caused by electron excitations.

**Figure 4:**
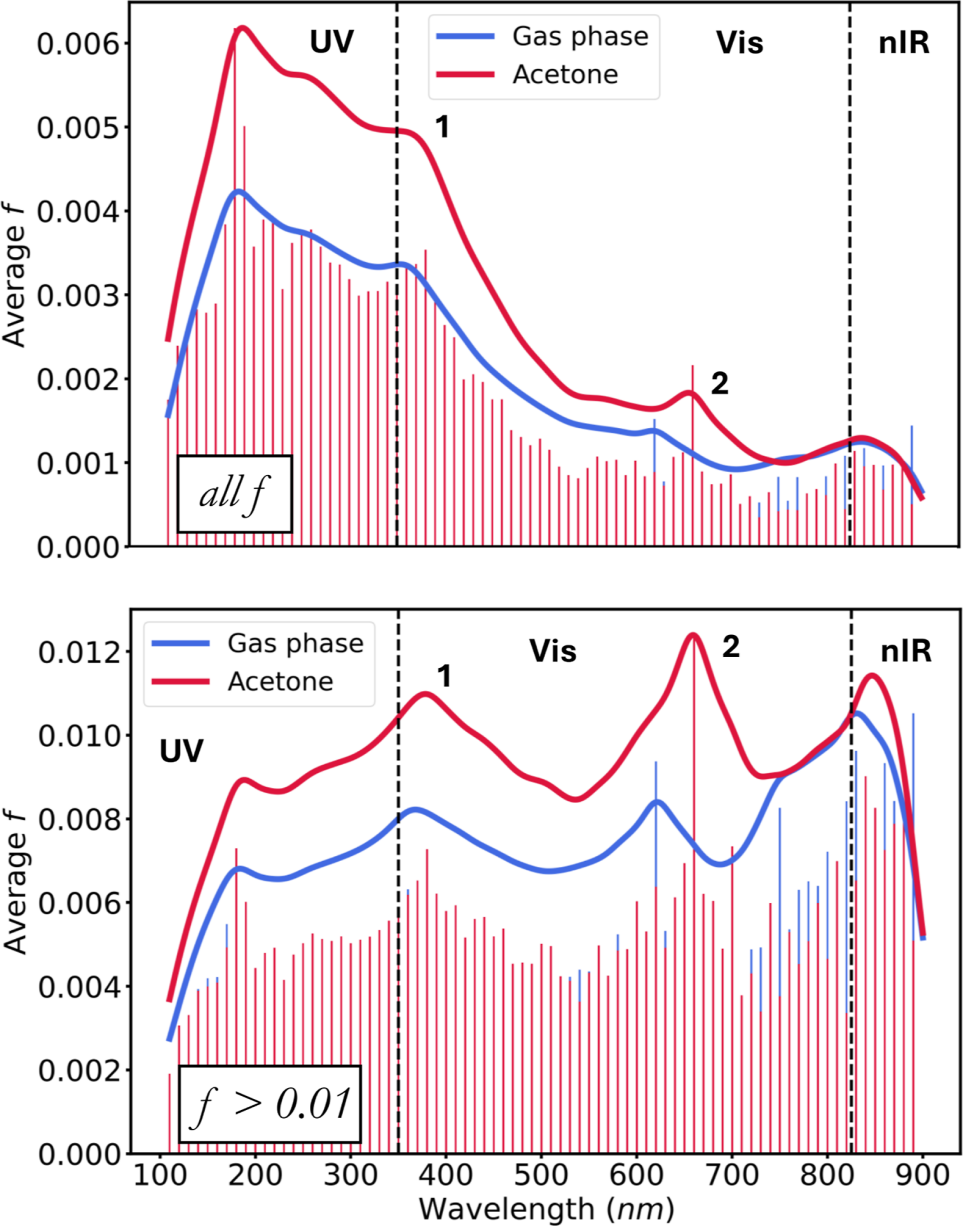
Averaged spectra over the whole data set for both the gas and acetone phases, considering either all excitations (top) or only those with *f* > 0.01 (bottom). The vertical dashed lines mark the limits between the UV, Vis, and nIR regions of the spectrum. The two Vis peaks are distinguished with the labels **1** and **2**. Curves were smoothened with a Lorentzian kernel.

Figure 5 illustrates the chemical diversity within the tmQMg* data set by showing ten examples of TMCs for which light absorption is maximal within the wavelength ranges defined by the four broad peaks of the gas phase *f* > 0.01 averaged spectrum (Figure 4). Both early and late transition metals from the 3d, 4d, and 5d series are present, in oxidation states within the {0, II, III, IV} set. There is also a total of 12 diverse ligands, which, in several cases, include different π-conjugated systems promoting absorption in the UV and Vis regions of the spectrum.

**Figure 5:**
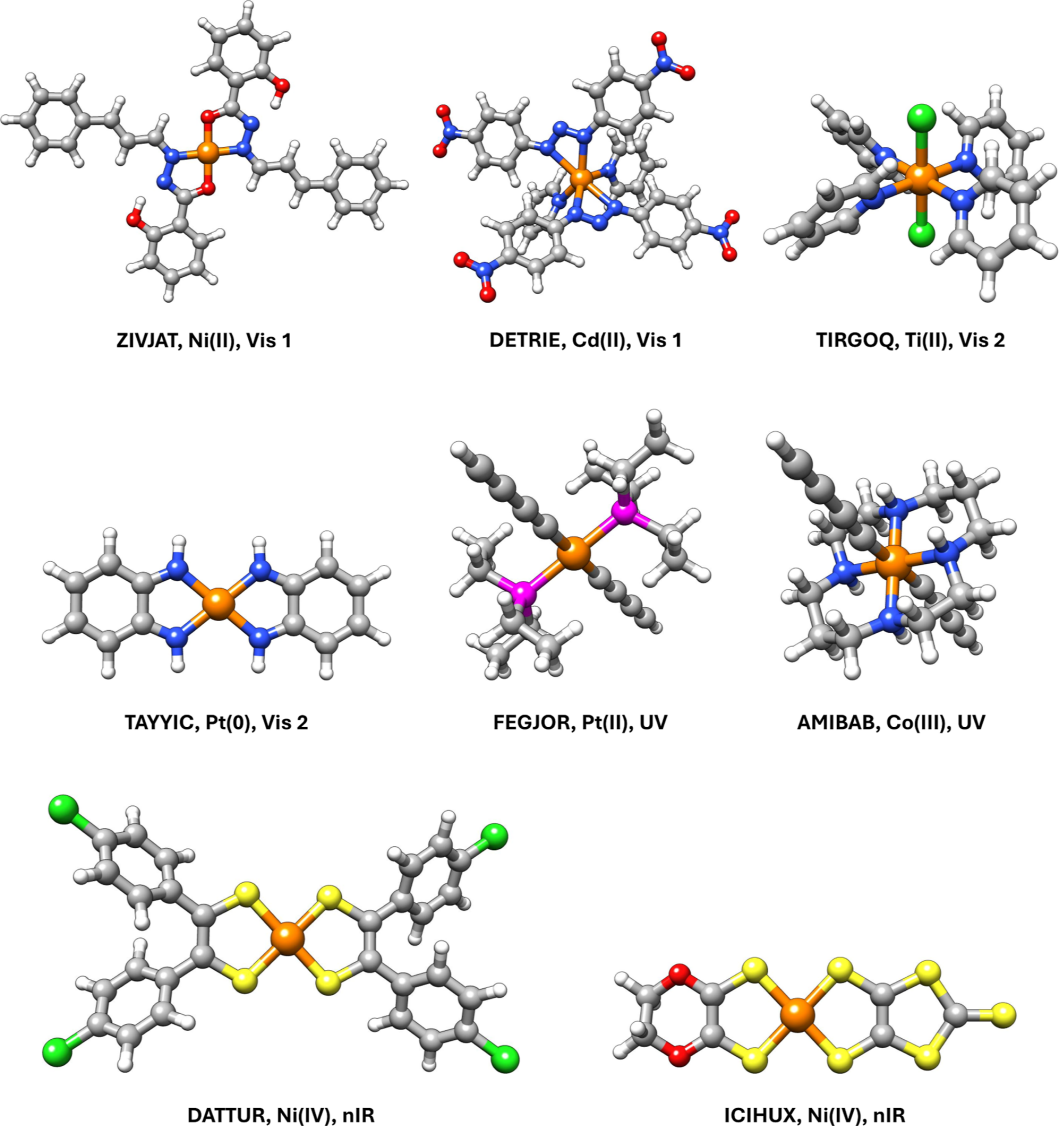
Examples of TMCs showing maximal light absorption in the ∼185 (**UV**), 375 (**Vis 1**), 645 (**Vis 2**), and 835 (**nIR**) nm peaks of the averaged *f* > 0.01 gas phase spectrum shown in Figure 4. Each TMC is also labeled with its 6-character CSD code and metal oxidation state. Element color code: orange metal, pink P, gray C, white H, blue N, red O, green Cl, and yellow S.

The electron excitations with the strongest intensity (*f*_max_) in the UV, Vis, and nIR ranges were also determined and included in the data set together with the corresponding wavelength (λ_max_; Table 1). For the TMCs that have no excitations, or no excitations with *f* > 0.01, no maximum absorption values are reported separately. Additionally, the band broadness, σ, was calculated for each of the three spectral regions, R , as

1 $$\sigma_{R}=\left|\Delta \lambda_{R}\right|\cdot n_{R}$$

where, for each R∈{UV,Vis,nIR} , $\left|\Delta \lambda_{R}\right|$ denotes the absolute difference between the smallest and largest wavelengths, and $n_{R}$ denotes the number of excitations within the $\left|\Delta \lambda_{R}\right|$ range.

All 17 properties in Table 1 are related to the diverse electronic structure space formed by the 74k TMCs in tmQMg* and, therefore, they appear correlated in different ways and to different extents. Figure 6 shows two examples of such correlations for the gas phase data. The plot of *f*_max_ versus HOMO–LUMO gap shows that the density of highly intense electron excitations decreases with the width of the gap, a trend that can be related to an increasing number of symmetry-forbidden *d* → *d*, *d* → π*, and π → *d* transitions. Further, the data appears stratified, with the nIR range shifted toward the smaller gaps, and the UV toward the larger gaps, with the Vis in the middle, in line with the excitation energy limits implicitly set by the HOMO–LUMO gap. In contrast, the plot of the natural metal charge against λ_max_ does not reflect any clear trend, in line with the lack of a strong correlation between these two properties, since both the metal and the ligand can have a strong influence on λ_max_.

**Figure 6:**
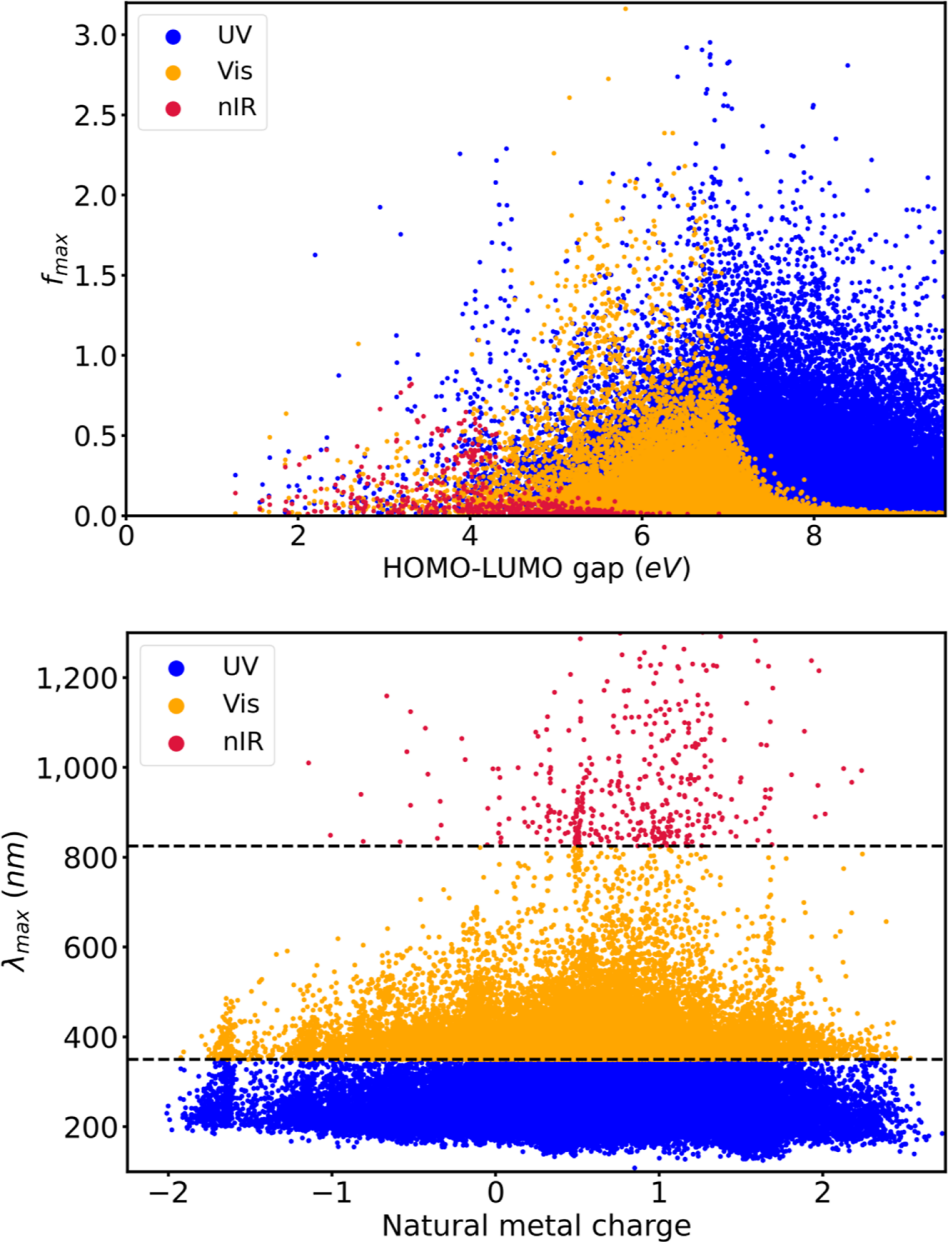
Scatter plots of the HOMO–LUMO gap (top) and natural metal charge (bottom) against the oscillator strength and wavelength, respectively, of the strongest excitations in the UV, Vis, and nIR regions of the spectrum, in gas phase. Metal charges, in *e* units, were taken from tmQMg, at the PBE0/def2TZVP level of theory.^53^

### Charge Transfer

For all TMCs in tmQMg* absorbing light in the visible range, NTO orbitals were computed for the most intense electron excitation at λ = λ_max_ ∈ Vis, with *f* = *f*_max_ > 0.01. The data defining the NTOs was used to classify these excitations as either a d-d orbital transition (ddT) at the metal center or one of these three charge transfer (CT) events: ligand-to-ligand (LLCT), metal-to-ligand (MLCT), and ligand-to-metal (LMCT). More precisely, we selected the pair of virtual (V) and occupied (O) NTOs having the largest eigenvalues (electron occupations) and, for both of them, we computed the ligands density, ρ­(*L*), resulting from summing the squared coefficients (*c* ^2^) of the natural atomic orbitals (in total, N_Orb_) over all atoms from all ligands in the TMC (in total, *N*_L_)­

2 $$\rho \left(L\right)=\sum_{i}^{N_{L}}\;\sum_{j}^{N_{Orb}}c_{i,j}^{2}$$

The same density was also computed for the metal alone, ρ­(*M*)­

3 $$\rho \left(M\right)=\sum_{j}^{N_{Orb}}c_{M,j}^{2}$$

Further, these densities were computed for both the V and O NTOs, overall yielding this set of values

4 $$\left\{\rho {\left(L\right)}_{O},\rho {\left(M\right)}_{O},\rho {\left(L\right)}_{V},\rho {\left(M\right)}_{V}\right\}$$

for each TMC. We finally computed the corresponding set of relative contributions

5 $$\left\{\rho {\left(L\right)}_{O}^{rel},\rho {\left(M\right)}_{O}^{rel},\rho {\left(L\right)}_{V}^{rel},\rho {\left(M\right)}_{V}^{rel}\right\}$$

using this expression

6 $$\rho {\left(\tau \right)}_{\gamma }^{rel}=\frac{\rho {\left(\tau \right)}_{\gamma }}{\rho {\left(M\right)}_{\gamma }+\rho {\left(L\right)}_{\gamma }}$$

where τ is either M or L, and γ is either V or O.

In order to define the **DA**CT labels of the charge transfer classes, where the donor (**D**) and acceptor (**A**), are either the metal or the ligand, the following relationships were used

7 $$D=\{\begin{array}{ll} Mif & \rho {\left(M\right)}_{O}^{rel}\geq 0.5,\\ Lif & \rho {\left(M\right)}_{O}^{rel}<0.5 \end{array}$$

8 $$A=\{\begin{array}{ll} Mif & \rho {\left(M\right)}_{V}^{rel}\geq 0.5,\\ Lif & \rho {\left(M\right)}_{V}^{rel}<0.5 \end{array}$$

For **D** = **A** = M, the resulting MMCT label was replaced by the more suitable ddT. It should be noted that the 0.5 threshold is used in a context in which the NTOs are rarely fully or strongly localized over the metal or the ligands alone but rather delocalized, to a variable extent, over these two structural moieties. For this reason, the tmQMg* data set also provides the NTO data of the electron excitations, allowing the users to modify the definition of the classes {ddT, LLCT, MLCT, LMCT} by simply adjusting the ρ­(*M*)_O_^rel^ and ρ­(*M*)_V_^rel^ thresholds.

Figure 7 shows the distribution of the different transition classes, all referring to the most intense transitions in the Vis range, for both the gas phase and acetone TD-DFT data. The small portion of ddT transitions can be related to their symmetry-forbidden nature, which prevents them from having strong intensities. LMCT and MLCT excitations are much more abundant and, together, they constitute nearly half of the excitations, in a proportion similar to that of the LLCT. The prevalence of the MLCT excitations over the LMCT may originate from the higher abundance of the late transition metals relative to the early (Figure 2). The increase in the number of both these excitations from the gas to the acetone phase can be related to the polarized nature of the metal–ligand bonds.

**Figure 7:**
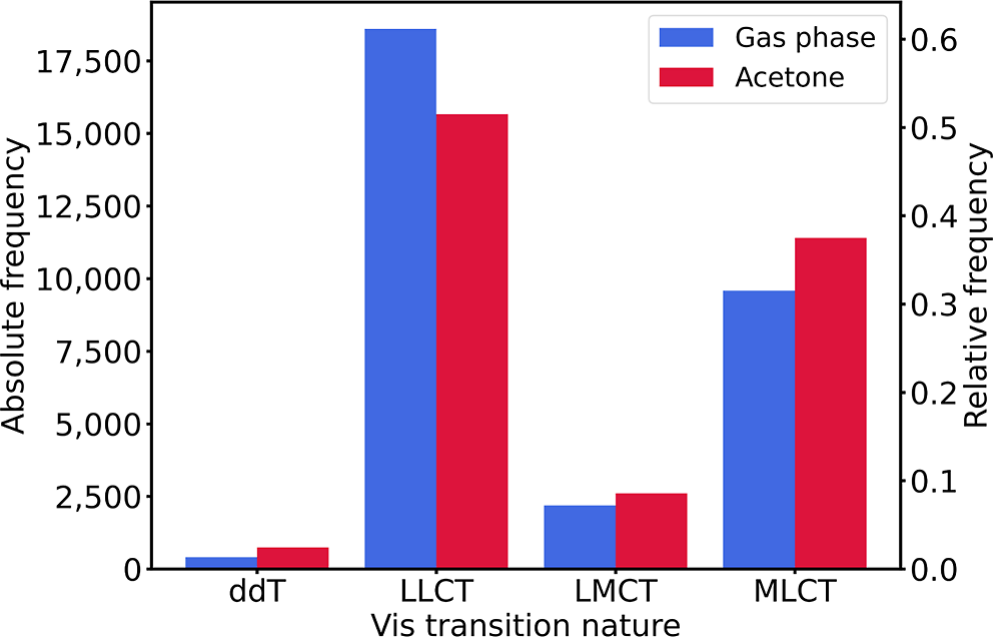
Distribution of the transition classes for the most intense electron excitations in the Vis range of the spectrum.

Figure 8 shows the average dipole moments and natural metal charges for each transition class in gas phase and acetone. The average dipole moments are systematically higher in acetone, as expected from the polarizing effects of the solvent. In both series, the dipole moment is similar regardless of the transition class, with only the two classes involving the metal, LMCT and MLCT, being slightly below and above the average, respectively, which could also be due to the polarized nature of the metal–ligand bonds. The average natural metal charges are quite similar in gas phase and acetone, with minor variations for the transitions involving the metal center. The transitions originating from the metal, that is, ddT and MLCT, are consistently associated with the smaller charges and thus the most electron-rich metal centers.

**Figure 8:**
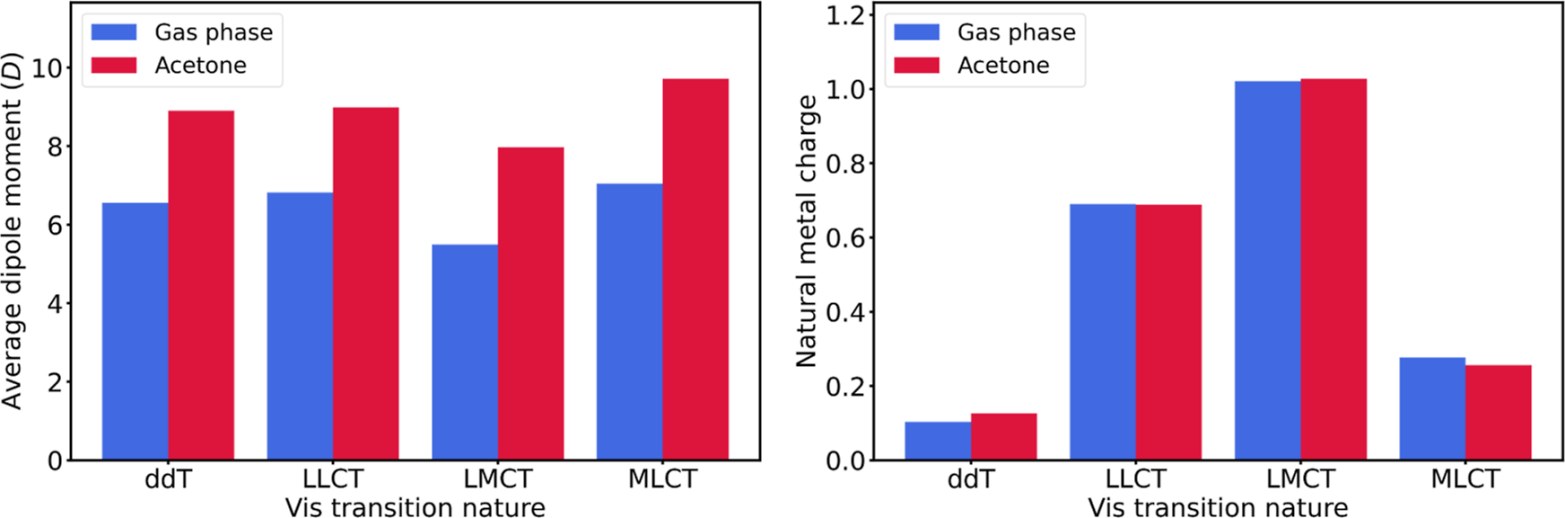
Average dipole moment (left) and natural metal charge (right) for the transition classes of the most intense electron excitations in the Vis range of the spectrum. The dipole moment was computed at the same TD-DFT level of theory, whereas the metal charges, in *e* units, at the PBE0/def2TZVP level, were taken from the tmQMg data set.^21^

### Solvatochromic Effects

The solvatochromism introduced by acetone relative to the gas phase, and observed in the average tmQMg* spectra shown in Figure 4, was quantified and reported in the data set after considering these four cases:

#### Case 1. Shift within the Visible

If a TMC absorbed light in the Vis range with *f* > 0.01 in both the gas and acetone phases, the solvatochromic effect was defined relative to the most intense electron excitations at λ = λ_max_ calculating both the wavelength shift

9 $$\Delta \lambda =\lambda_{\max,Vis}^{ac}-\lambda_{\max,Vis}^{gp}$$

and the change in the associated oscillator strength, *f*_λ_max,Vis__

10 $$\Delta f=f_{\lambda_{\max,Vis}}^{ac}-f_{\lambda_{\max,Vis}}^{gp}$$

where the ‘gp’ and ‘ac’ superscripts denote the gas and acetone phases, respectively.

In this case, the vis-to-vis property of Table 1 was set to True, whereas the uv-to-vis, NIR-to-vis, vis-to-uv, and vis-to-NIR properties were all set to False.

#### Case 2. Shift to the Visible

If a TMC absorbed light in the Vis range with *f* > 0.01 but only in the acetone phase, the most intense electron excitation defined by $\left(\lambda_{\max,Vis}^{ac},f_{\lambda_{\max,Vis}}^{ac}\right)$ was selected and used to calculate the solvatochromic effects relative to the most intense electron excitation in the gas phase in either the UV or nIR regions, selecting the one with the closest wavelength if both existed; that is

$$\Delta \lambda =\{\begin{array}{ll} \lambda_{\max,Vis}^{ac}-\lambda_{\max,nIR}^{gp} & if\;\left|\lambda_{\max,Vis}^{ac}-\lambda_{\max,nIR}^{gp}\right|<\left|\lambda_{\max,Vis}^{ac}-\lambda_{\max,UV}^{gp}\right|\\ \lambda_{\max,Vis}^{ac}-\lambda_{\max,UV}^{gp} & if\;\left|\lambda_{\max,Vis}^{ac}-\lambda_{\max,nIR}^{gp}\right|>\left|\lambda_{\max,Vis}^{ac}-\lambda_{\max,UV}^{gp}\right| \end{array}$$ 11

and

$$\Delta f=\{\begin{array}{ll} f_{\lambda_{\max,Vis}}^{ac}-f_{\lambda_{\max,nIR}}^{gp} & if\;\left|\lambda_{\max,Vis}^{ac}-\lambda_{\max,nIR}^{gp}\right|<\left|\lambda_{\max,Vis}^{ac}-\lambda_{\max,UV}^{gp}\right|\\ f_{\lambda_{\max,Vis}}^{ac}-f_{\lambda_{\max,UV}}^{gp} & if\;\left|\lambda_{\max,Vis}^{ac}-\lambda_{\max,nIR}^{gp}\right|>\left|\lambda_{\max,Vis}^{ac}-\lambda_{\max,UV}^{gp}\right| \end{array}$$ 12

In Table 1, either the NIR-to-vis or uv-to-vis properties were set to True, respectively, while setting all others to False.

#### Case 3. Shift Away from the Visible

If a TMC absorbed light in the Vis range with *f* > 0.01 but only in the gas phase, the most intense electron excitation defined by $\left(\lambda_{\max,Vis}^{gp},f_{\lambda_{\max,Vis}}^{gp}\right)$ was selected and used to calculate the solvatochromic effects relative to the most intense electron excitation in the acetone phase in either the UV or nIR regions, selecting the one with the closest wavelength if both existed; that is

$$\Delta \lambda =\{\begin{array}{ll} \lambda_{\max,nIR}^{ac}-\lambda_{\max,Vis}^{gp} & if\;\left|\lambda_{\max,nIR}^{ac}-\lambda_{\max,Vis}^{gp}\right|<\left|\lambda_{\max,UV}^{ac}-\lambda_{\max,Vis}^{gp}\right|\\ \lambda_{\max,UV}^{ac}-\lambda_{\max,Vis}^{gp} & if\;\left|\lambda_{\max,nIR}^{ac}-\lambda_{\max,Vis}^{gp}\right|>\left|\lambda_{\max,UV}^{ac}-\lambda_{\max,Vis}^{gp}\right| \end{array}$$ 13

and

$$\Delta f=\{\begin{array}{ll} f_{\lambda_{\max,nIR}}^{ac}-f_{\lambda_{\max,Vis}}^{gp} & if\;\left|\lambda_{\max,nIR}^{ac}-\lambda_{\max,Vis}^{gp}\right|<\left|\lambda_{\max,UV}^{ac}-\lambda_{\max,Vis}^{gp}\right|\\ f_{\lambda_{\max,UV}}^{ac}-f_{\lambda_{\max,Vis}}^{gp} & if\;\left|\lambda_{\max,nIR}^{ac}-\lambda_{\max,Vis}^{gp}\right|>\left|\lambda_{\max,UV}^{ac}-\lambda_{\max,Vis}^{gp}\right| \end{array}$$ 14

In Table 1, either the vis-to-NIR or vis-to-UV properties were set to True, respectively, while setting all others to False.

#### Case 4. No Shift

If a TMC did not exhibit any electron excitation in the Vis range with *f* > 0.01 in neither the gas nor the acetone phases, both the wavelength shift and the intensity change were set to zero.

15 Δλ=0

16 Δf=0

In this case, in Table 1, vis-to-vis = uv-to-vis = NIR-to-vis = vis-to-NIR = vis-to-uv = False. This also includes a few instances derived from Cases 2 and 3, in which Δλ and Δ*f* could not be defined due to inexistent transitions with *f* > 0.01 in neither the UV nor the nIR ranges.

With these definitions of Δλ and Δ*f*, the bathochromic, hypsochromic, hyperchromic, and hypochromic properties of Table 1 were set to either True or False based on the relationships defined in Table 2. Figure 9 shows the proportional distributions of these properties in tmQMg*, also providing this information for Cases 1–4. Whereas approximately half of the TMCs in the data set have no activity in the Vis range, and thus no solvatochromism, the other half mostly undergo bathochromic and hyperchromic effects.

**Table 2:** Criteria Defining the Solvatochromism Properties in Table 1

criteria	bathochromic	hypsochromic
Δλ > 0	true	false
Δλ < 0	false	true

criteria	hyperchromic	hypochromic
Δ*f* > 0	true	false
Δ*f* < 0	false	true

**Figure 9:**
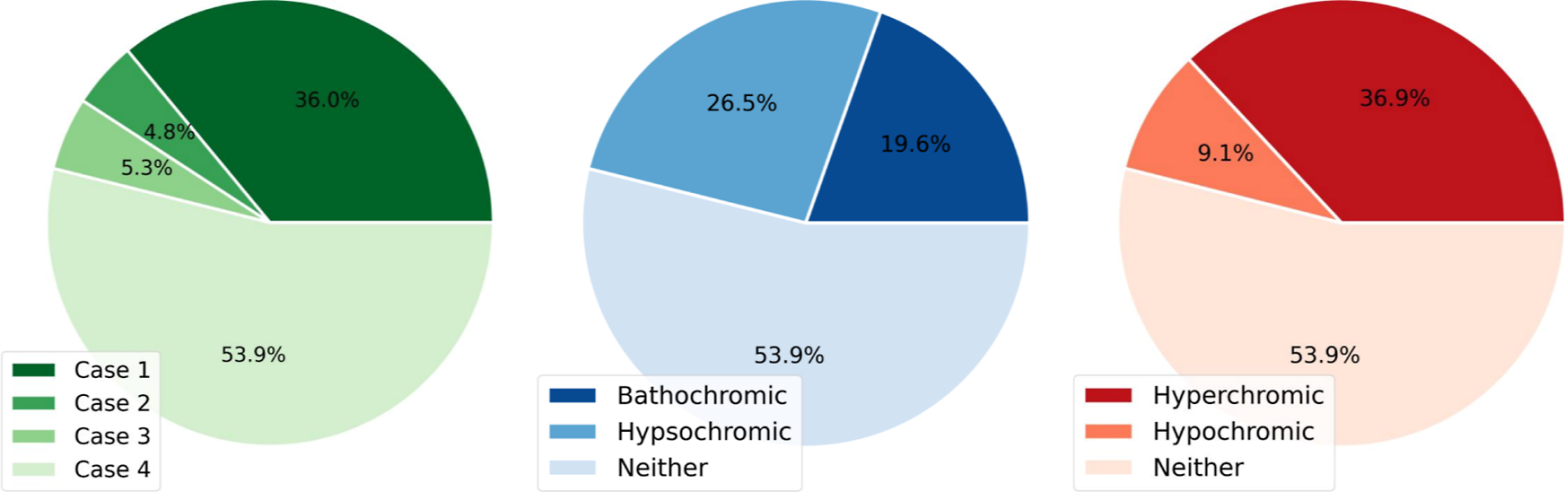
Pie charts showing the relative % amounts of TMCs in Cases 1–4, and exhibiting batho- and hypsochromic effects, as well as hyper- and hypochromic effects.

Figure 10 shows the scatter of Δ*f* versus Δλ for all TMCs in tmQMg* subject to the solvatochromic effects defined by Cases 1–3. The data point density shows that many TMCs exhibit solvatochromic effects with Δ*f* and Δλ values clustered within the approximate ranges [−0.25, +0.50] and [−100, +100] nm, respectively. There is thus a prevalence of the hyperchromic effect over the hypochromic, whereas the bathochromic and hypsochromic shifts are more balanced, in line with the pie charts shown in Figure 9. Δ*f* and Δλ appear weakly correlated. Nearby the origin and within the region having the highest data point density, either the intensity becomes larger with Δλ ≈ 0 or, orthogonally, the wavelength becomes shorter or longer with Δ*f* ≈ 0. There are also two significantly dense regions in the opposing (Δλ > 0, Δ*f* < 0) and (Δλ < 0, Δ*f* > 0) quadrants, suggesting a weak correlation in which Δλ increases while Δ*f* decreases.^54^

**Figure 10:**
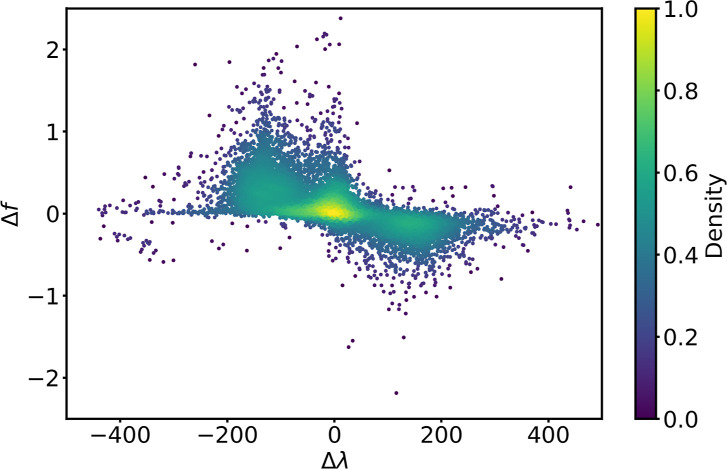
Solvatochromic effects in tmQMg* in (Δλ, Δ*f*) coordinates. The density values were determined with a Gaussian kernel, using the min–max normalization over the [0, 1] range.

## Conclusion

The present work introduced the tmQMg* data set, which contains excited state properties computed at the TD-DFT­(ωB97xd/def2SVP) level for 74,281 TMCs, all known in the CSD database and reflecting the wide diversity of the metal–organic space known to date from crystallography. The data set provides information regarding the maximum absorption wavelength and band broadness within the UV, Vis, and nIR ranges of the spectrum. Further, the nature of the charge transfer in the most intense electron excitation in the Vis range is included, giving also the metal and ligand contributions to the associated NTO orbitals. All data was computed for both the gas and acetone phases, yielding the solvatochromic effects on the wavelengths and oscillator strengths, which are also provided. The tmQMg* data set will enable further data-driven studies on the photochemistry of the transition metals, including the development of discriminative ML models for the prediction of absorption spectra and generative ML models for the de novo design of novel TMC chromophores. The level of theory used in this work, TD-DFT­(ωB97xd/def2SVP), was chosen aiming at a reasonable balance between cost and accuracy, given the large size of the data, 74k. Other functionals and basis sets, for example, ωB97M-V^55^ and def2TZVP,^46^ should, in principle, provide higher accuracy but also at a much higher cost. From this perspective, the present work could motivate the development of Δ-ML models for correcting TMC excited state properties toward higher levels of theory. Conversely, the tmQMg* data set can be used to benchmark lower levels of theory like, for example, semiempirical methods using the Tamm-Dancoff approach.

## Supplementary Material



## Data Availability

All data is openly available at 10.5281/zenodo.17209767 and additional analysis code is provided at https://github.com/uiocompcat/tmQMg_star.

## Notes

Details on failed TD-DFT calculations, averaged spectra using different oscillator strength thresholds, averaged spectra by metal series, and nature of the electron excitations in the Vis range by transition metal series (PDF)
