# Supplementary material for: tmQMg* Data Set: Excited State Properties of 74k Transition Metal Complexes
Source: J Chem Inf Model. 2025 Oct 24;65(21):11766–77. doi: 10.1021/acs.jcim.5c01958 (PMC12606629; doi:10.1021/acs.jcim.5c01958)
Supplement: Supplementary file 1 [file ci5c01958_si_001.pdf]

Supporting Information for tmQMg* Dataset: Excited State Properties of 74k Transition Metal Complexes

Hannes Kneiding,[†] David Balcells[†,*]

[†]*Hylleraas Centre for Quantum Molecular Sciences, Department of Chemistry, University of Oslo, P.O. Box 1033, Blindern, 0315 Oslo, Norway*

E-mail: david.balcells@kjemi.uio.no

Table of Contents

Failed TD-DFT calculations, page S3

Averaged spectra using different oscillator strength thresholds, page S5

Averaged spectra by metal series, page S6

Vis transition nature by metal series, page S7

Failed TD-DFT calculations

Overall, the TD-DFT calculations failed to converge for 159 and 202 TMCs in gas phase and acetone, respectively. With an overlap of 107 TMCs, for which both calculations failed, there were 254 unique TMCs for which the calculation in gas phase or acetone failed. All failed calculations exited with the Gaussian error message *Excessive mixing of frozen core and valence orbitals* suggesting a breakdown of the frozen core approximation for these compounds. Among the failed TMCs, the distribution of non-metal elements appears to be similar to the distribution observed for the rest of the dataset, with the exception of an increased presence of oxygen (Figure S1). However, the distribution of metal atoms shows a dramatically increased failure rate for TMCs with Yttrium or Lanthanum metal centers (Figure S2), pointing to their shallow core-valence separation as the most probable cause of the TD-DFT calculation failure.

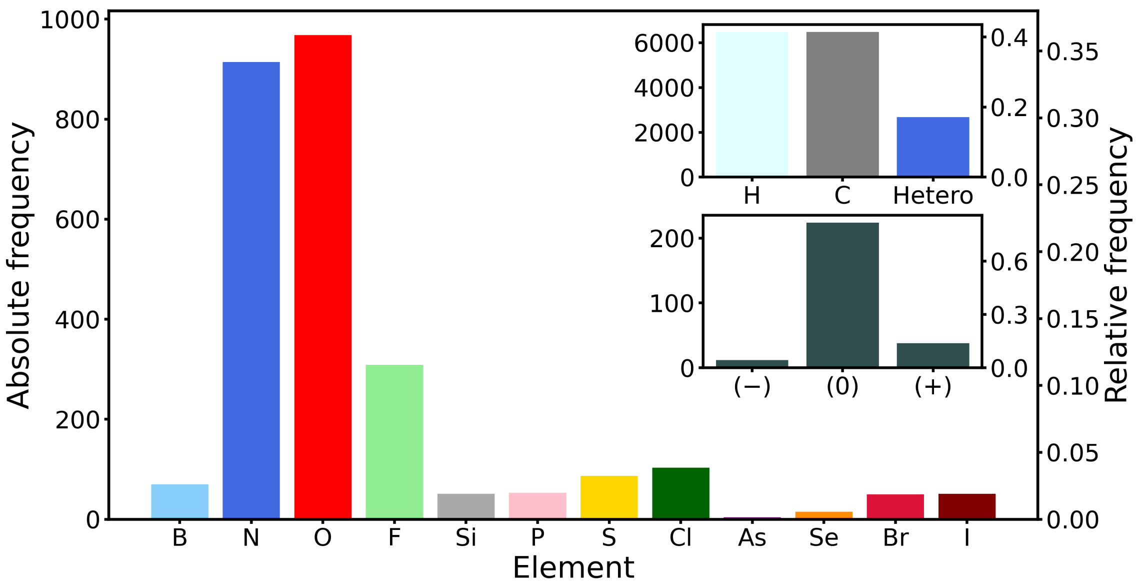

Figure S1: Distribution of the non-metal elements in the failed TMCs. The insets show the abundance of C and H relative to the hetero-elements, and the TMC charge distribution.

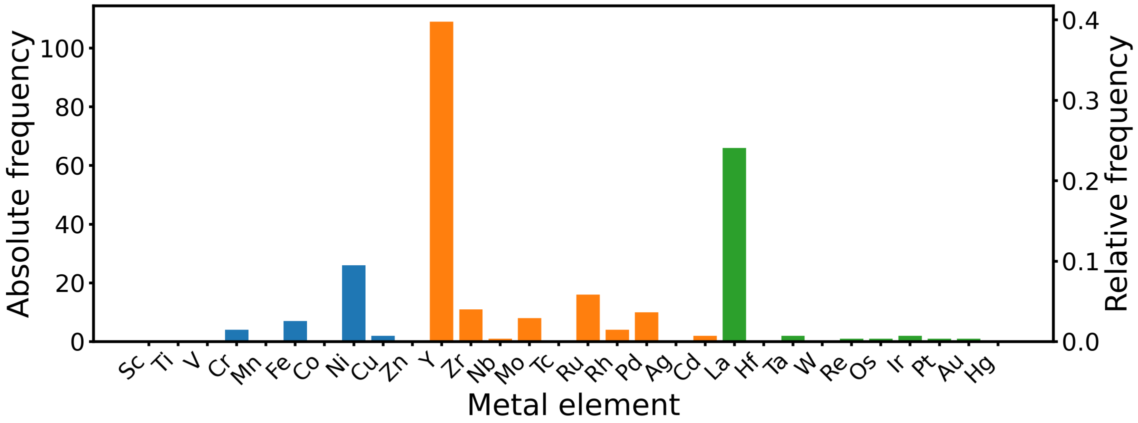

Figure S2: Distribution of the transition metal elements in the failed TMCs.

Additionally, there were 20 unique TMCs (10 in gas phase, 15 in acetone, and 5 in both) yielding negative oscillator strength values in the TD-DFT output that were excluded, yielding a total of 274 excluded TMCs from the parent tmQMg dataset.

Averaged spectra using different oscillator strength thresholds

Figure S3 shows averaged spectra using additional oscillator strength thresholds (0.001 and 0.1). Qualitatively, the averaged spectra, including the peak positions, seem to be fairly robust to larger oscillator strength cutoffs. However, as the threshold is decreased, the resulting spectra quickly start exhibiting a dominant shoulder in the UV range similar to the average spectra obtained using no threshold for the oscillator strength. This is due to the fact that the TD-DFT calculations produce a large number of low intensity excitations in the UV range.

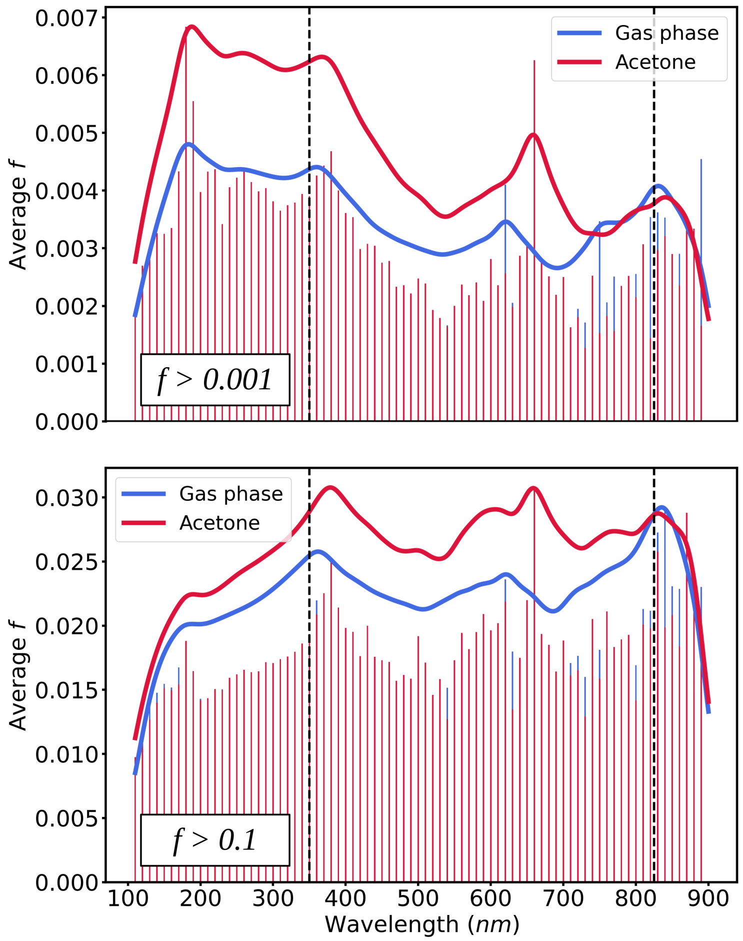

Figure S3: Averaged spectra over the whole dataset for both the gas and acetone phases, considering excitations with $f > 0.001$ (top) or only those with $f > 0.1$ (bottom). The vertical dashed lines mark the limits between the UV, Vis, and nIR regions of the spectrum. Curves were smoothened with a Lorentzian kernel.

Averaged spectra by metal series

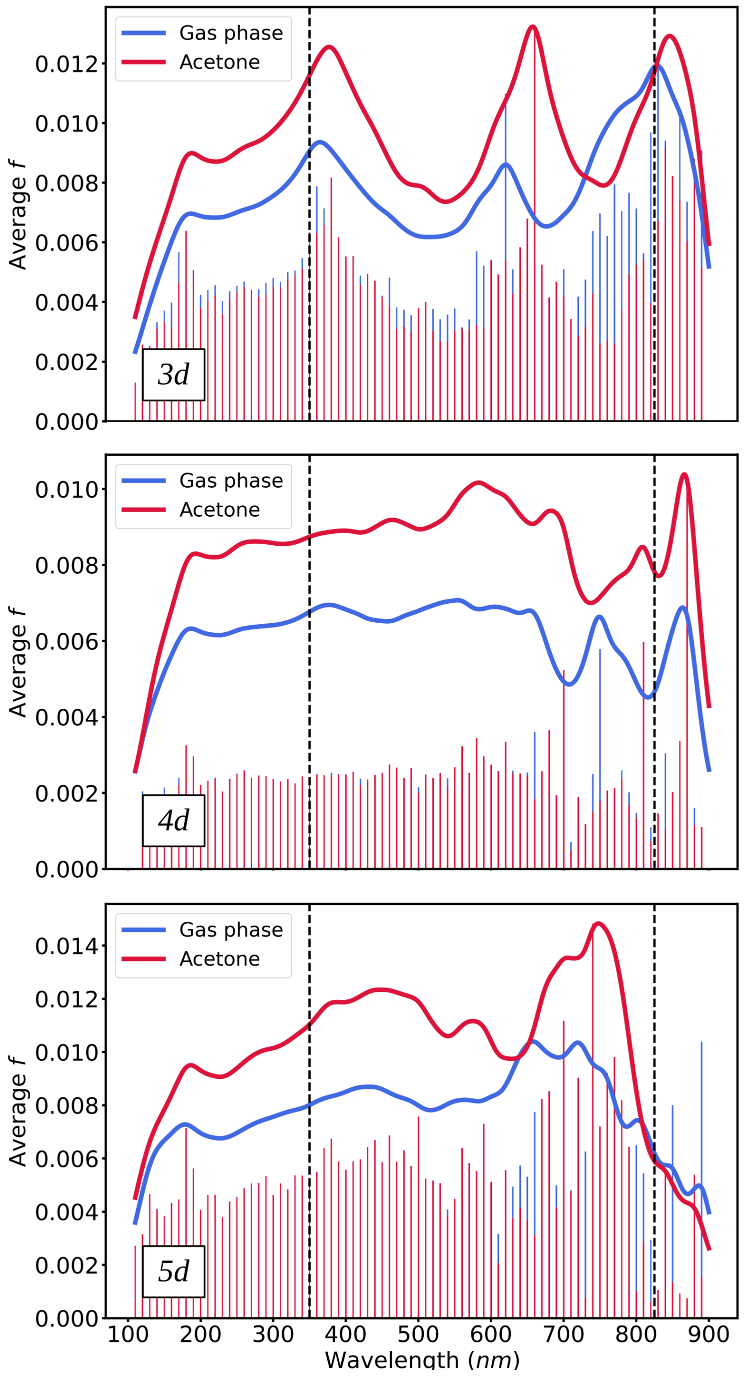

Figure S4: Averaged spectra over subsets of the dataset corresponding to the TMCs with metal centers from the 3*d*, 4*d* and 5*d* series, respectively, for both the gas and acetone phases, considering excitations with $f > 0.01$. The vertical dashed lines mark the limits between the UV, Vis, and nIR regions of the spectrum. Curves were smoothened with a Lorentzian kernel.

Vis transition nature by metal series

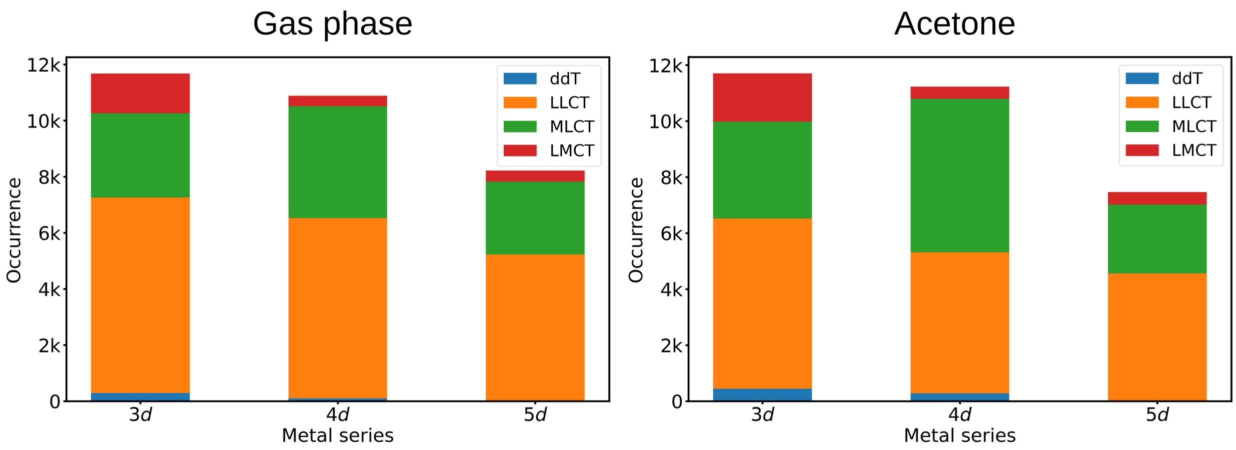

Figure S5: Occurrence of the different Vis transition natures in TMCs with metal centers from the 3d, 4d and 5d series, respectively, for both the gas and acetone phases.
